# Recovery of SARS-CoV-2 from Wastewater Using Centrifugal Ultrafiltration

**DOI:** 10.3390/mps4020032

**Published:** 2021-05-12

**Authors:** Brienna L. Anderson-Coughlin, Adrienne E. H. Shearer, Alexis N. Omar, K. Eric Wommack, Kalmia E. Kniel

**Affiliations:** 1 Center for Environmental and Wastewater-Based Epidemiological Research, Department of Animal and Food Sciences, University of Delaware, Newark, DE 19716, USA; briennaa@udel.edu (B.L.A.-C.); ashearer@udel.edu (A.E.H.S.); aomar@udel.edu (A.N.O.); 2 Center for Environmental and Wastewater-Based Epidemiological Research, Department of Plant and Soil Sciences, University of Delaware, Newark, DE 19716, USA; wommack@udel.edu

**Keywords:** SARS-CoV-2, wastewater, ultrafiltration, RT-qPCR, COVID-19 surveillance

## Abstract

The COVID-19 pandemic is a global crisis and continues to impact communities as the disease spreads. Clinical testing alone provides a snapshot of infected individuals but is costly and difficult to perform logistically across whole populations. The virus which causes COVID-19, SARS-CoV-2, is shed in human feces and urine and can be detected in human waste. SARS-CoV-2 can be shed in high concentrations (>10^7^ genomic copies/mL) due to its ability to replicate in the gastrointestinal tract of humans through attachment to the angiotensin-converting enzyme 2 (ACE-2) receptors there. Monitoring wastewater for SARS-CoV-2, alongside clinical testing, can more accurately represent the spread of disease within a community. This protocol describes a reliable and efficacious method to recover SARS-CoV-2 in wastewater, quantify genomic RNA levels, and evaluate concentration fluctuations over time. Using this protocol, viral levels as low as 10 genomic copies/mL were successfully detected from 30 mL of wastewater in more than seven-hundred samples collected between August 2020 and March 2021. Through the adaptation of traditional enteric virus methods used in food safety research, targets have been reliably detected with no inhibition of detection (RT-qPCR) observed in any sample processed. This protocol is currently used for surveillance of wastewater systems across New Castle County, Delaware.

## 1. Introduction

Human wastewater has the potential to be contaminated with pathogens of various etiologies including bacteria, parasites, and viruses. Methods to evaluate wastewater contamination and the efficacy of water remediation treatments generally focus on detection and enumeration of human enteric microorganisms including fecal coliform bacteria and enteric viruses as well other human fecal indicator viruses such as pepper mild mottle virus (PMMoV) [1]. However, wastewater-based epidemiology (WBE) has also been employed for human pathogens in cases there was interest in the viral pathogens in wastewater itself rather than in the contamination of an environmental water source. The presence and/or levels of these organisms can help to monitor outbreak progression or, conversely, the dissemination of vaccines within a population. During the 2013 norovirus (NoV) and hepatitis A virus (HAV) outbreaks in Scandinavia, three unique HAV strains were detected, two of which were linked to clinical cases; NoV detection preceded clinical case occurrence by up to three weeks [2]. Throughout the Poliomyelitis epidemics of the twentieth and twenty-first centuries, poliovirus (PV) serotypes I-III were monitored and compared to the poliovirus vaccines, live oral (OPV) or inactivated (IPV), Sabin-strains. In Japan, all three serotypes of PV were detected in human wastewater between 2010 and 2013 along with the OPV and IPV strains [3]. Likewise, a fourteen-year study was performed in Moscow, Russia, between 2004 and 2017 in which all three serotypes were detected in at least one of the 5450 human wastewater samples collected throughout that time [4]. These studies highlight the importance of wastewater surveillance and have been successfully implemented alongside clinical measures during the poliomyelitis vaccination transitional period as well as through Russia’s National Poliomyelitis Eradication Program, established in 1996.

Although SARS-CoV-2 is a respiratory viral pathogen, its genomic material has been detected in fecal material [5,6], and its presence in human wastewater may serve as an early indicator of viral shedding within communities. Coronaviruses and enteric pathogens or their indicator organisms vary considerably in structural features important for establishing experimental protocols for their recovery and quantification. Protocols for recovering virus from wastewater involve various filtration and concentration steps to separate virus from larger microorganisms and solids [1,5] that could limit downstream detection of viral genomic material. SARS-CoV-2 is an enveloped, single-stranded RNA virus. The diameter of coronaviruses ranges from 60 to 220 nm [7], with SARS-CoV-2 ranging in size from 60 to 140 nm in diameter [8]. The closely related SARS-CoV-1 is spherical or ellipsoidal in shape, with an average diameter of 80 to 90 nm [9]. Regarding other microorganisms of interest in human wastewater, SARS-CoV-2 is smaller than the enteric bacterium *Escherichia coli,* which is approximately 1 µm (1000 nm), but larger than enteric viruses, norovirus [10], and hepatitis A virus [11], which are approximately 30 nm in diameter. Thus, current filtration testing protocols designed to recover enteric viruses from wastewater samples based on size-exclusion are applicable to recovery of SARS-CoV-2.

SARS-CoV-2 differs from the aforementioned enteric pathogenic viruses, however, in that it has a lipid outer envelope [9]. The hydrophobicity of the viral envelope could impact viral recovery with potential for greater viral adherence to solids and greater susceptibility to degradation by solvents and detergents in wastewater as compared to nonenveloped viruses [7]. Prior studies demonstrated that concentration and filtration protocols involving PEG precipitation, acidic adsorption, and alkaline elution commonly used for enveloped viruses may have comparatively lower recovery rates for bovine, porcine, and murine coronaviruses [5,12,13,14,15], whereas ultrafiltration improved coronavirus recovery [15]. Ultrafiltration with membranes of molecular weight cut offs of 1 to 100 kDa were previously demonstrated to remove enteric viruses from water [1]. A review of the limited data on coronavirus recovery from wastewater and environmental water concluded that robust methods are needed for concentrating coronaviruses and other enveloped viruses from large volumes of various types of water [5]. 

The protocol presented herein describes SARS-CoV-2 recovery and detection from human wastewater, as adapted from traditional virus detection methods used in food and water safety research. The method is based on size-exclusive filtration of wastewater through a hydrophilic, low-protein binding 0.22 µm polyethersulfone membrane to capture solids, protozoa, and most bacteria but allow passage of virus into the filtrate. This membrane filtration step is followed by viral capture and concentration with a 10 kDa molecular size-exclusion membrane via centrifugal ultrafiltration. Subsequent nucleic acid extraction, detection, and quantification through RT-qPCR molecular assay were performed and microbial concentrations per liter of wastewater were determined. The reliable and efficient quantification of SARS-CoV-2, among other organisms of concern, from wastewater provided by the adoption of this method allows for widespread and consistent monitoring of populations to be performed.

The methods described here were designed to be highly versatile for use across various laboratory environments, levels of expertise, and organisms of interest. There is no use of ultracentrifugation equipment or cell culture assays which require BSL-3 facilities for work with SARS-CoV-2. Moreover, a single sample can be processed to target multiple organisms, and automated nucleic acid extraction techniques and disposable vacuum filter houses can be replaced with manual extraction and filter membranes in re-usable labware apparatuses. The adaptability of this protocol will allow it to be of continued use with the global infrastructure established through COVID-19 research after the conclusion of the pandemic.

## 2. Experimental Design

This protocol was developed to optimize the recovery and detection of SARS-CoV-2 from wastewater. Methodologies that were created for enteric virus surveillance of agricultural irrigation waters and other environmental sources were adapted to account for the differences in physicochemical properties of wastewater compared to other water sources as well as the morphological differences between non-enveloped enteric viruses and the enveloped SARS-CoV-2. Wastewater was composited over 24-h-periods, collected, and transported to the Center for Environmental and Wastewater-based Epidemiological Research (CEWER) (University of Delaware, Newark, DE, USA) for processing. Samples were collected from the general population, University of Delaware campus housing facilities, and primary and secondary schools (wastewater composited during school hours, not 24-h collection) throughout New Castle County, DE. 

SARS-CoV-2 N1 and N2 genes were detected and quantified using real-time RT-qPCR. SARS-CoV-2 detection levels were compared to standard curves generated from a commercial plasmid control (IDTDNA), and the concentrations of viral copies per liter of wastewater were calculated. Concentrations below the limit of quantification (LOQ) of 10,000 viral copies/L were reported as not detected. More than seven-hundred samples have been processed to date (August 2020–March 2021), with SARS-CoV-2 levels ranging between less than the LOQ to approximately 10^7^ viral copies/L. Viral concentrations above the LOQ were analyzed by averaging the N1 and N2 detection data for replicates of each sample. Data were compiled and displayed publicly by New Castle County, which can be viewed at: https://compassred.shinyapps.io/ncco_wastewater/ (accessed 5 October 2021).

Pepper mild mottle virus (PMMoV) was used as an environmental process control in this study. PMMoV is widely used as a fecal indicator and has been implemented as a normalization organism to determine the relative fecal concentration of wastewater due to the high concentrations of the virus shed in human feces [16,17,18]. Tulane virus (TV) is a commonly employed methodological process control for enteric virus research and was detected alongside SARS-CoV-2 and PMMoV in the initial method development of this study [19]. Nuclease-free water was used as a negative control in molecular detection to ensure there was no contamination of the reagents, proper efficiency of the primers and probes, and amplification of target genes were above the established threshold.

### 2.1. Materials

#### 2.1.1. Wastewater Collection

- Disposable polypropylene centrifuge tubes, 50 mL (Thermo Fish Scientific, Waltham, MA, USA; Cat. no.: 05-539-13)

#### 2.1.2. Wastewater Incubation

- Kimwipes delicate task wipers 1-ply (Kimberly-Clark, Irving, TX, USA; Cat. no.: 06-666)
- 75% ethanol
- 50 mL tube racks

#### 2.1.3. Wastewater Filtration

- Filter system, 150 mL, 0.22 µm PES membrane (Corning Inc., Corning, NY, USA; Cat. no.: 431153)

#### 2.1.4. Virus Concentration

- Amicon Ultra-15 centrifugal filters (Merck Millipore, Carrigtwohill, CO, USA; Cat. no.: UFC901096D)

#### 2.1.5. Nucleic Acid Extraction

- QIAamp Viral RNA Mini Kit (Qiagen, Hilden, Germany; Cat. no.: 52906)
- Rotor adapters (Qiagen; Cat. no.: 990394)
- Filter-tips 1000 µL (Qiagen; Cat. no.: 990352)

#### 2.1.6. Molecular Detection (RT-qPCR)

- Quantinova Probe RT-PCR Kit (Qiagen; Cat. no.: 990352)
- Strip tubes and caps, 0.1 mL (Qiagen; Cat. no.: 981103)
- SARS-CoV-2 CDC RUO primers and probes (Integrated DNA Technologies, Coralville, IA, USA; Cat. no.: 10006713)
- 2019-nCoV_N_positive control (Integrated DNA Technologies; Cat. no.: 10006625)

### 2.2. Equipment

#### 2.2.1. Wastewater Collection

- Portable sampler unit (Teledyne ISCO, Lincoln, NE, USA; Cat. no.: 6837000069 and 699003588)

#### 2.2.2. Wastewater Incubation

- Bead bath dry bath (Lab Armor LLC, Irving, TX, USA; Cat. no.: SL-74309-720)

#### 2.2.3. Wastewater Filtration

- Class II biosafety cabinet (Labconco, Kansas City, MO, USA; Cat. no.: 36208)

#### 2.2.4. Virus Concentration

- Allegra X-12R tabletop centrifuge (Beckman Coulter, Brea, CA, USA; Cat. no.: 392472)

#### 2.2.5. Nucleic Acid Extraction

- Digital vortex mixer (Thermo Fish Scientific, Waltham, MA, USA; Cat. no.: 02-215-418)
- QIAcube Connect automated extraction unit (Qiagen; Cat. no.: 9002864)

#### 2.2.6. Molecular Detection (RT-qPCR)

- PCR Workstation (CBS Scientific, East Lyme, CT, USA; Cat. no.: P-030-202)
- Rotor-Gene Q 2-Plex system apparatus (Qiagen; Cat. no.: 9001620)

## 3. Procedure

This protocol is designed for the recovery and detection of SARS-CoV-2 and other microbiological organisms (e.g., Pepper Mild Mottle Virus (PMMoV), crAssphage, and Bacteroides) of interest from wastewater. 

### 3.1. Wastewater Collection. Time for Completion: 24:00 h

1. Collect wastewater at hourly intervals manually or through programming a portable sampler unit over a twenty-four-hour period. If wastewater is collected from a location which is only occupied during specific hours (e.g., schools or businesses), then collect during that time only.
2. Thoroughly mix the composited wastewater, then transfer 40–45 mL into two 50 mL centrifuge tubes and tightly cap the tubes.
3. Transport samples to the laboratory and decontaminate the exterior of the tubes using 75% ethanol, or equivalent.

### 3.2. Wastewater Incubation. Time for Completion: 01:00 h

**Scheme 1:**
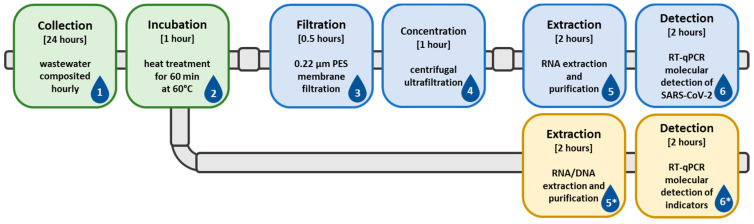
Visual representation of the wastewater sample collection and processing scheme. The scheme begins with collection (1) and incubation (2), then continues into filtration (3), viral concentration (4), nucleic acid extraction (5), and molecular detection (6). Additional steps (5* and 6*) can be added after the incubation (2) period, for detection of normalization or indicator organisms.

1. Invert the sample tubes several times to suspend settled solids then place the tubes in the bead bath, which is pre-heated to 60 °C.
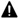
**CRITICAL STEP** Incubation should be performed to not only decrease the risks of processing human wastewater but also to homogenize the sample and keep the solids in suspension while processing. Greater recovery (*p* < 0.05; data not shown) of SARS-CoV-2 was observed after incubation was performed.
2. Incubate the wastewater for thirty minutes, invert tubes to re-suspend settled solids, then return the samples to the bead bath for an additional thirty minutes.
3. After incubation for the full sixty minutes is completed, remove the samples from the bead bath.
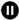
**PAUSE STEP** Allow wastewater to cool by storing samples at refrigeration temperature (4 °C) prior to performing filtration (Step 3.3). Water above ambient temperature may impact the PES membrane of the filter unit. Samples may be stored up to 72 h at 4 °C before continuing the processing; however, concentration should be completed to reach the frozen storage stage without exceeding this timeframe.
    **OPTIONAL STEP** Remove aliquots (>1 mL) of incubated wastewater for detection of normalization organisms (Scheme 1, Steps 5* and 6*). Detection of additional organisms can be performed using the method continued below; however, considerations then need to be made to account for variations in recovery of the organisms due to their morphological differences.

### 3.3. Wastewater Filtration. Time for Completion: 00:30 min

1. Transfer 40–45 mL of wastewater from one tube into a 0.22 µm Corning filter unit.
2. Connect the filter to a vacuum system and allow the wastewater to pass through the membrane until a minimum of 30 mL can be collected as filtrate and the large particles have been removed.
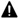
**CRITICAL STEP** Filtration of the wastewater is necessary to remove particles which may cause inefficient concentration; increased turbidity and volume of viral concentrates (3.4) were observed when filtration was omitted. Additionally, nucleic acid extraction (3.5) efficiency may decrease or inhibition of molecular detection (3.6) may occur.

### 3.4. Virus Concentration. Time for Completion: 01:00 h

1. Collect 30 mL of filtrate and divide evenly between two Amicon 10 kDa centrifugal filters.
2. Place the Amicon filters into the centrifuge and spin for 45 min at 3000× *g* at ambient temperature.
3. Collect the viral concentrate from the two filters, and pool the concentrate for each sample in a microcentrifuge tube.
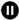
**PAUSE STEP** The protocol may be paused here by storing viral concentrates at −20 °C until nucleic acid extraction is performed. If samples are frozen, they must be thawed to room temperature prior to nucleic acid extraction.

### 3.5. Nucleic Acid Extraction. Time for Completion: 02:00 h

1. Perform extractions in duplicate for each sample using QIAamp Viral RNA Mini Kit and the QIAcube automated extraction instrument per the manufacturer’s instructions.
  **OPTIONAL STEP** Nucleic acid extractions may be performed manually in lieu of using the automated system by following the manufacturer’s instructions.
  **OPTIONAL STEP** RNA and DNA of additional target organisms can be extracted simultaneously using Qiagen’s RNeasy PowerMicrobiome Kit (Cat. no.: 26000-50). Recovery efficacy of other virus, bacteria, or phage should be monitored separately from SARS-CoV-2 due to morphological differences. Larger organisms should be processed directly after incubation (3.2) or eluted from the filter (3.3).
  (a) Add a filter column from the QIAamp kit to each rotor adapter in position 1 and 1.5 mL collection tube from the Rotor Adapter set in position 3 then place the Rotor Adapters into the centrifuge of the QIAcube.
  (b) Prepare the Buffer AVL-carrier RNA solution (560 µL Buffer and 5.6 µL RNA per sample) and add 560 µL to two microcentrifuge tubes for each sample.
  (c) Pipette to mix the virus concentrate and add 140 µL to two tubes containing the Buffer AVL-carrier RNA solution.
  (d) Pulse-vortex the samples for 15 s and incubate at room temperature for 10-min.
  (e) While samples are incubating, transfer 700 µL from the microcentrifuge tubes to CB tubes.
  (f) Place the CB tubes in the QIAcube and follow the on-screen instructions for the QIAamp Viral RNA Mini Kit, adjusting the elution volume to 50 µL and keeping all other options at the manufacturer’s recommended settings.
2. Remove the Rotor Adapters from the QIAcube centrifuge when the run is complete and place in a biosafety cabinet.
3. Discard the filter column, close the collection tube and transfer to a microcentrifuge tube rack. Discard the waste in the bottom of the Rotor Adapter into a flammable waste container for chemical disposal.
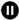
**PAUSE STEP** The protocol may be paused here by storing nucleic acids at −20 °C until detection is performed. If samples are frozen, prior to detection they must be thawed on ice before adding RNA template to the RT-qPCR reaction. If long-term storage of RNA is required, transfer to −80 °C.

### 3.6. Molecular Detection (RT-qPCR). Time for Completion: 02:00 h

1. Allow reagents, and nucleic acids if necessary, to thaw.
2. Prepare the reaction mix using 10 µL of Master Mix, 0.2 µL of Reverse Transcriptase Mix, 1 µL of each primer and probe, and water to a total volume of 17 µL per reaction tube. Primers and probes should be at 8000 and 4000 nM stock concentrations, respectively to achieve the concentrations shown in Table 1 for the reaction mix.
3. Transfer 17 µL of the reaction mix to each tube, then add template RNA, negative and positive controls.
4. Cap the strip tubes and place in the Rotor-Gene Q.
5. Program the instrument to run at 45 °C for 10 min, 95 °C for 5 min, and 40 cycles of 95 °C for 5 s and 60 °C for 30 s. Set to acquiring during the 60 °C period for both green (FAM) and yellow (HEX) channels.

### 3.7. Data Interpretation and Analyses

1. After the RT-qPCR run is complete, perform the in-system analysis by setting values for the standard curve or importing an existing curve, then setting the threshold.
2. Export the data and calculate the average viral copies/L using the N1 and N2 target copies/reaction data. Report data with no amplification above the threshold as below the limit of quantification or limit of detection, which for this protocol is 10,000 copies/L.

## 4. Expected Results

RT-qPCR data from positive detection results above the limit of quantification should be analyzed; values which are obtained and below the limit of quantification should not be included in data analyses. Results from this study of more than seven-hundred samples ranged from below the limit of quantification (10^4^ copies/L) to greater than 10^7^ copies/L. RT-qPCR data are relatively quantified, compared to digital droplet PCR (ddPCR) assays which provide absolute quantification. This limitation is commonly experienced as ddPCR is a relatively new technology that is still limited in availability and requires additional method development for use with these types of samples. Care should be taken when comparing data across laboratories to ensure they are appropriately represented, and claims are justified. Additionally, in smaller populations the identification of new SARS-CoV-2 infections is complicated by the presence of recovering individuals within the population. Due to the limited data available at this time, the shedding rates of newly infected and recovered individuals and the timeframe of shedding remains unknown. Moreover, the impacts of population demographics including age, vaccination rates, and re-infection are unknown and may increase the complexity of data analysis as the pandemic progresses. Before the vaccinations and variant surges in Delaware began, the analyses of SARS-CoV-2 wastewater concentrations from large populations was performed using the baseline, established when case levels were low, and evaluating the increasing and decreasing trends. Greater fluctuation of SARS-CoV-2 concentrations observed in smaller populations means that adjustments for population size, wastewater flow rates, and housing and plumbing situations need to be implemented for greater understanding of the data. Moving forward, changes are expected in SARS-CoV-2 concentrations and the relationship with COVID-19 clinical cases as vaccination rates and variant strains of the virus develop. Adjustments to analyses will need to be considered as the data is evaluated.

## 5. Reagents Setup

Primers and probes are diluted to the necessary concentrations (Table 1) per the manufacturer’s (IDTDNA) instructions using IDTE pH 8.0.

## Figures and Tables

**Table 1:** Primer and probe sequence and concentration information for use with RT-qPCR. SARS-CoV-2 primers and probes target two regions, N1 and N2, which require two primer sets that are used concurrently in duplex RT-qPCR reactions [20].

Target	Sequence (5′-3′) ^1^	Conc. (nM)
N1	F: GAC CCC AAA ATC AGC GAA AT	400
	R: TCT GGT TAC TGC CAG TTG AAT CTG	400
	P: FAM-ACC CCG CAT TAC GTT TGG TGG ACC-BHQ1	200
N2	F: TTA CAA ACA TTG GCC GCA AA	400
	R: GCG CGA CAT TCC GAA GAA	400
	P: FAM-ACA ATT TGC CCC CAG CGC TTC AG-BHQ1	200

^1^ The F, R, and P proceeding the sequence indicate that it is used as the Forward (F) and Reverse (R) primers, and probe (P). SARS-CoV-2: N1 and PMMoV were detected using FAM labeled probes on the green detection channel, and SARS-CoV-2: N2 was detected using the HEX labeled probe on the yellow detection channel.

## Data Availability

Data are made publicly available through New Castle County, DE at https://compassred.shinyapps.io/ncco_wastewater/ (accessed 5 October 2021).
